# The effect of aquatic training and vitamin D3 supplementation on bone metabolism in postmenopausal obese women

**DOI:** 10.1016/j.jesf.2024.01.002

**Published:** 2024-01-12

**Authors:** Foroozandeh Zaravar, Gholamhossein Tamaddon, Leila Zaravar, Maryam Koushkie Jahromi

**Affiliations:** a General Sciences Department, School of Paramedical Sciences, Shiraz University of Medical Sciences, Shiraz, Iran; b Division of Hematology and Blood Bank, Department of Laboratory Sciences, School of Paramedical Sciences, Shiraz University of Medical Sciences, Shiraz, Iran; c Department of Sport Sciences, School of Education and Psychology, Shiraz University, Shiraz, Iran

**Keywords:** VitaminD3, Aquatic exercise, Bone metabolism, Menopause

## Abstract

**Purpose:**

Despite prevalence of studies indicating the positive effect of land-based exercise on bone metabolism, there are limited findings regarding the effect of aquatic exercise. The present study aimed to evaluate the effects of aquatic training and vitamin D3 supplementation on femur bone mineral density (BMD), serum 25(OH)D, and parathyroid hormone (PTH) in postmenopausal obese women with vitamin D insufficiency.

**Methods:**

40 postmenopausal obese women were randomly divided into four groups of aquatic training + vitamin D3 intake group; (ATD), aquatic training with placebo intake group (AT), vitamin D3 intake group (D), and control group with placebo intake (CON). AT groups performed aerobic aquatic exercises for 8 weeks. Vitamin D3 supplementation groups consumed oral dose of 4000 IU/d for 8 weeks.

**Results:**

The femur BMD was significantly higher in the ATD than the AT and D and CON groups; in AT it was higher than the D and CON groups. Serum 25(OH)D level in the ATD was more than AT and CON, and in the D was more than the CON and AT. PTH in the ATD group was lower compared to AT, D, and CON groups. PTH was lower in the AT and D compared to the CON.

**Conclusion:**

In postmenopausal obese women with vitamin D insufficiency or deficiency, combining vitamin D supplementation and aquatic training was the most effective method for improving bone metabolism; Vitamin D supplementation (alone) was not sufficient to affect some of bone metabolism indices; Aquatic training could not improve serum vitamin D. By priority, ATD, AT, and D indicated better bone related metabolism indices.

## 1 Introduction

Osteoporosis is the most prevalent metabolic bone disease in the world in the way that the frequency of osteoporosis increases from 1/3 in people at age 50–60 to more than 50 % in people aged over 80 years.^1^ Peak bone mass is achieved in the mid-20 to 40 years for different bones. Subsequently, bone mass normally declines, so that at the age of 70 years, bone mass decrease by 30–40 %. The frequency of osteopenia and osteoporosis is much higher in women compared to men.^2^ In postmenopausal women, osteoporosis-induced fractures are more frequent than stroke, myocardial infarction, and breast cancer and these fractures can be very expensive and result in disability or death.^3^ Abrupt reduction of estrogen during menopause can be an important effective factor in inducing osteoporosis.^4^ Also, it has been found that one-third of the postmenopausal women with osteoporosis had increased parathyroid hormone (PTH) levels.^5^

Several studies have confirmed the inverse relationship between PTH, and bone mineral density (BMD).^6^^,^^7^ During bone resorption, PTH mediates the expression of the receptor activator of nuclear factor κ-B ligand (RANKL), engaged in osteoclast formation, through the PKA pathway.^8^^,^^9^ However, some studies in animal models indicated a beneficial effect of PTH on BMD.^10^^,^^11^ Various environmental factors impact BMD, PTH and bone metabolism.^12^ Exercise is one of the environmental factors that contribute to the process of increasing peak bone mass.^4^ It is shown that inactivity or sedentary behavior increases bone loss during menopause,^13^ while muscle contractions even induced by electricity cause strain reactions in bone.^14^ Most of the previous findings indicated that only land-based weight-bearing and resistance training induce a positive effect on bone density.^15^^,^^16^

There are controversies regarding the effect of swimming on bone health. Some studies indicated that swimmers had lower BMD compared to sedentary people and complementary exercises such as jumping rope or whole-body vibration are recommended to them for increasing their bone mineral density.^17^ It has been suggested that swimming and synchronized swimming should be combined with weight-bearing, impact, or strength activities.^18^ However, water-based exercises include muscle contraction and seem to induce beneficial effects on osteogenesis. A meta-analysis study indicated that water-based exercise was beneficial for maintaining or improving bone health in postmenopausal women but was less beneficial when compared to land-based exercise, while emphasizing the discrepancies in findings and the necessity of future research for more clarifications.^19^ Exercise also stimulates the release of parathyroid hormone (PTH).^12^ In both humans and animals, the transient increase in PTH levels is dependent on the type, intensity, and duration of exercise^20^^,^^21^ that could be accompanied by increasing trabecular bone volume along with cortical bone volume and modulus of elasticity.^22^ A review study found that vitamin D intake led to a decrease in PTH levels, while exercise led to an increase in PTH,^12^ while the effect of combined exercise and vitamin D intake on PTH is not clear yet.

It is clear that water-based exercises are recommended to older people due to their physical limitations, chronic musculoskeletal pain and stiffness^23^ and its benefits for neuromuscular, and functional fitness.^24^ Another important problem of older people is vitamin D deficiency which can be related to various health problems including low BMD. This deficiency may even not be treated by just vitamin D supplementation.^25^ So, it seems that another involvement such as exercise can be recommended to compensate for this deficiency. A study indicated that weight bearing outdoor compared to weight bearing indoor exercises would have the combined benefits of physical activity and sun exposure and increased the synthesis of vitamin D.^26^ However, the increased plasma concentration of vitamin D occurs with physical activity both indoors and outdoors.^27^ A study indicated that moderate swimming is beneficial in improving vitamin D status in diabetic patients.^28^ No study was found regarding the effect of water-based exercise on serum vitamin D in postmenopausal women. Vitamin D can also be related to PTH function and their interaction affects BMD.^29^ So, this study aimed to evaluate the effect of vitamin D supplementation with or without aquatic exercise on serum 25(OH)D, PTH and BMD.

## 2 Research method

### 2.1 Design and participants

This was an experimental study which was conducted during late autumn and winter. 65 postmenopausal women who did not participate in regular Physical exercise before the study aged 60–70 years took part in the study voluntarily. To recruit the participants, an announcement was distributed in different pension offices with the information about the research procedures, inclusion criteria, and aims. Then, 45 volunteer women who were eligible according to inclusion criteria were selected. Finally, according to exclusion criteria, 40 women (ages: 65.65 ± 3.29 years, BMI:44.66 ± 5.96 kg/m^2^) were selected for data analysis. Participants were from similar socioeconomic status.

Inclusion criteria were the age range of 60–70 years, at least 10 years following the last menstrual period, not using vitamin D supplements currently or for the three months prior to the experiment, vitamin D insufficiency or lower range sufficiency, not suffering from metabolic and hormonal diseases affecting the study variables, not taking any high kind of hormonal therapy, not smoking, not having participated in any kind of exercise for at least five months prior to the study. Exclusion criteria were unwillingness to continue participating the study, not participating regularly on exercise programs related to the study (maximum of three absences in exercise sessions was allowed) or not taking vitamin D supplements as required by the study and not suffering from any kind of disease affecting the research variables.

The present study followed the ethical recommendation according to the Declaration of Helsinki (1975, revised in 1983). Informed consent was obtained from all participants before participation in any treatment and they signed related form. The study proposal and procedures was approved by the graduate and Ethics Committee (https://methics.sums.ac.ir/page-Methics/fa/170/form/pId24912, of Shiraz University of Medical Sciences (No: IR.SUMS.REC.1400.466).

### 2.2 Procedures

Participants in treatment and control groups were referred to the same bone density medical centers and the pathology laboratory to assess BMD by dual-energy x-ray absorptiometry (DXA) in the femur area and to take blood samples to assess serum 25(OH)D, and parathyroid hormone. The weight and height of participants were measured using a scale-stadiometer (Seca-755) and Body Mass Index (BMI) was calculated by wight(kg)/height(m).^2^ Participants were randomly divided into four groups: (1) the aquatic training group + vitamin D3 intake (ATD), (2) the aquatic training group without vitamin D3 intake and placebo intake (AT), (3) the vitamin D3 intake group (D), and (4) control group with placebo intake (CON). To observe ethical considerations, following treatment periods, aquatic exercise facilities were provided for non-exercise groups and vitamin D3 supplements were recommended and provided for the non-vitamin D group. It should be noted that during the 12 weeks, participants were recommended not to change their routine diet or activity and not to use any supplement other than vitamin D in related groups. Also, the overall health condition (e.g. blood pressure, pulse, breathing, temperature, pain, and any unusual symptoms) of all 4 groups was monitored by a physician every 15 days.

Both training groups (AT and ATD) trained for 12 weeks, 3 sessions per week and 60 min per session. The first 10 min of each session included stretching and flexibility exercises and very slow walking in the water to warm up. During the main exercise program, in the first and second week, participants spent 40 min on special aerobic exercises, including brisk walking and aerobic movements in water, endurance exercises with water resistance to increase cardiovascular endurance and muscle strength, especially in the lower limbs, as well as improving balance. The intensity of exercise was measured by the Borg scale (1–10 score) which was about 5–6 in the first two weeks and increased to 6 to 7 in the last weeks. The intensity, duration, and repetition of exercise increased progressively. During the recovery or cooling down phase, participants performed 10 min of slow walking and very light stretching movements and floating in the water.

Participants were selected from insufficient serum 25(OH)D (20–30 ng/ml) or lower-level sufficient serum 25(OH)D (30–35 ng/ml). So according to one study finding^30^ and physician recommendation, oral dose of 4000 IU/d was prescribed for them for 2 months to vitamin D3 groups. Placebo intake groups consumed placebo pills containing 1 g of wheat flour. *Biochemical assessments*.

24 h before the first experiment session and 24 h after the last experiment session, 5 ccs of blood were taken from the brachial vein of the participants following 12 h of night fasting. Blood samples were centrifuged at a speed of 3000 rpm for 20 min and the serum was separated by a pipette. Serum 25-hydroxyvitamin D [25(OH)D] concentration was measured based on the commercial kit Elecsys Vitamin D Total II (Roche Diagnostics GmbH). The ECLIA automated electrochemiluminescence method was performed by the COBAS analyzer (the cobas e411 System). PTH was measured using commercial reagent kits from Roche Diagnostics GmbH.

Statistical data analysis was performed using SPSS software version 21. Results were presented as mean ± standard deviation (SD). The Shapiro-Wilk test was used to test for determining the normality of the distribution of data. Regarding the normality of findings, the analysis of covariance (ANCOVA) test was used to compare posttests while the pretest was considered as a covariate. In the case of significant difference, the LSD test was used to compare paired groups.

## 3 Results

Out of 45 volunteers, 40 postmenopausal women ages 65.65 ± 3.29 year [ATD (65.100 ± 3.212), AT (65.100 ± 3.478), D (66.000 ± 3.559), CON(66.400 ± 3.238)] were included in the study data analysis. They were categorized as overweight or obese as their BMI was 45.63 ± 5.96 kg/m^2^[ATD (45.640 ± 7.985), AD (45.156 ± 5.876), D (46.816 ± 5.956), CON (44.927 ± 4.817)]. There was no significant in BMI before intervention in four groups. After the interventions [ATD (43.008 ± 7.72), AT (42.53 ± 5.20), D (46.67 ± 5.17) and CON (45.36 ± 4.77)], the groups were compared using ANCOVA test, while pre-test was defined as covariate, which indicated lower BMI in ATD(p < 0.001) and AT(p < 0.005) groups compared to CON and D groups. There were no significant differences between other groups.

Considering main study variables including BMD which was 0.62 ± 0.07 gr/cm^2^ [ATD (0.585 ±0 .080), AT (0.554 ±0 .051), D (0.517 ±0 .062), CON (0.571 ±0 .033)], they were recognized with osteopenia or osteoporosis. Their serum vitamin D(OH) was 27.54 ± 6.23 ng/ml [AT (28.340 ± 5.427), AD (27.990 ± 6.540), D (25.770 ± 7.456), CON (28.080 ± 5.995)] which can be categorized to insufficient or borderline serum vitamin D. Their basal PTH was 65.02 ± 14.61(pg/ml) [(ATD (66.940 ± 13.608), AT (64.990 ± 14.516), D (63.940 ± 16.272), CON (64.210 ± 16.097)] which can be considered as borderline or increased PTH(>65 pg/ml) (Table 1). Comparison of variables between groups, before interventions, indicated that there was no significant difference in serum vitamin D, and PTH of four groups before the interventions. While, BDM in ATD and AT was higher than D and CON groups(p < 0.05).

**Table 1:** Comparisons of study variables between groups after the intervention program using ANCOVA and considering pretest as covariate.

		Pre-intervention Mean (SD)	Post-intervention Mean (SD)	F	P
BMD (gr/cm^2^)	Aquatic training + vitamin D	.585(.080)	0.707(0.082)	8.444	*<0.001
	Aquatic training	.554(.051)	0.647(0.066)		
	Vitamin D	.517(.062)	0.573(0.033)		
	Control	.571(.033)	0.536(0.036)		
Serum 25(OH)D (ng/ml)	Aquatic training + vitamin D	28.340(5.427)	46.840(13.304)	45.456	<0.001*
	Aquatic training	27.990(6.540)	20.440(5.737)		
	Vitamin D	25.770(7.456)	51.030(11.050)		
	Control	28.080(5.995)	20.700(5.851)		
PTH (pg/ml)	Aquatic training + vitamin D	66.940(13.608)	31.430(13.715)	70.971	*P < 0.001
	Aquatic training	64.990(14.516)	59.310(16.018)		
	Vitamin D	63.940(16.272)	63.160(16.881)		
	Control	64.210(16.097)	78.750(7.746)		

BMD: bone mineral density; PTH: parathyroid hormone, *significant difference(p < 0.05).

The ANCOVA test was used to compare post intervention BMD in the studied groups, while pre-intervention BMD was defined as covariate. According to the findings, there was a statistically significant difference in post-intervention BMD (post) between the different groups (F = 5.567, p = 0.003) (Table 1).

**Fig. 1:**
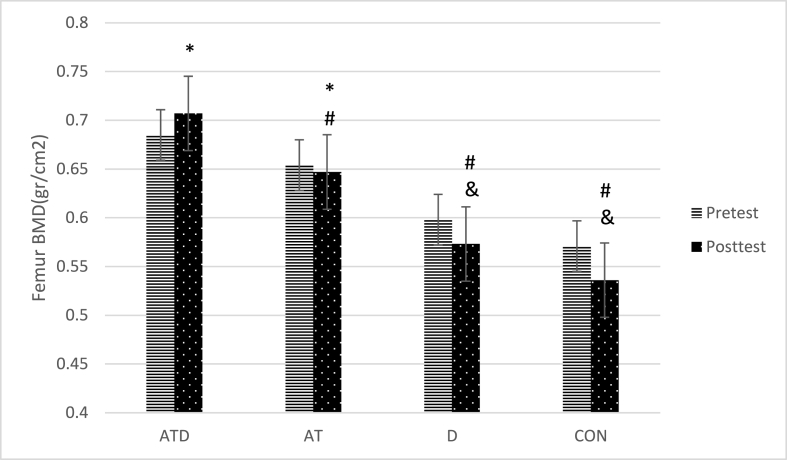
comparison of femur BMD in study groups, * significant difference with CON group, # significant difference with ATD group, & significant difference with AT group. Aquatic training group + vitamin D intake group (ATD), Aquatic training group (AT), vitamin D intake group (D), control group (CON).

The comparison of pairs of groups as indicated in Table 1 and Fig. 1 showed that femur BMD was significantly higher in ATD than the AT (p = 0.012) and D (p = 0.01) and CON (p = 0.01) groups. Also, BMD in the AT was higher than the D (p = 0.013) and CON (p = 0.007) groups. There was no significant difference between other paired groups(p > 0.05).

Regarding serum vitamin D, ANCOVA was used to compare groups, and pretest vitamin D was included as a covariate. As shown in Table 1, findings indicate a significant difference between groups (F = 45.456, p < 0.001).

**Fig. 2:**
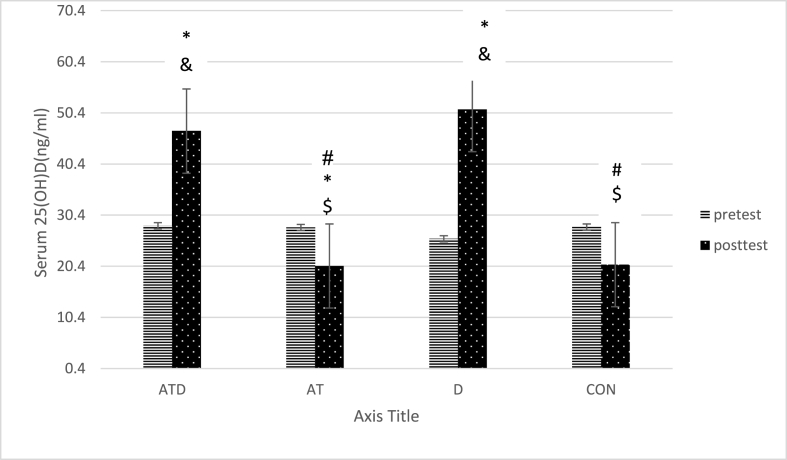
Comparison of serum 25(OH)D in the study groups, * significant difference with CON group, # significant difference with ATD group, & significant difference with AT group, $ significant difference with D group. Aquatic training group + vitamin D intake group (ATD), Aquatic training group (AT), vitamin D intake group (D), control group (CON).

Paired group comparisons indicated that the serum 25(OH)D level in the ATD was more than AT and CON (p < 0.001), the serum 25(OH)D level in the D was more than the CON and AT (p < 0.001) (Table 1, Fig. 2). There was no significant difference between other paired groups(p > 0.05).

Regarding serum PTH, ANCOVA test was used to compare groups, and pretest PTH was defined as covariate. As indicated in Table 1 significant difference was found in PTH between groups (F = 70.971, p < 0.001).

**Fig. 3:**
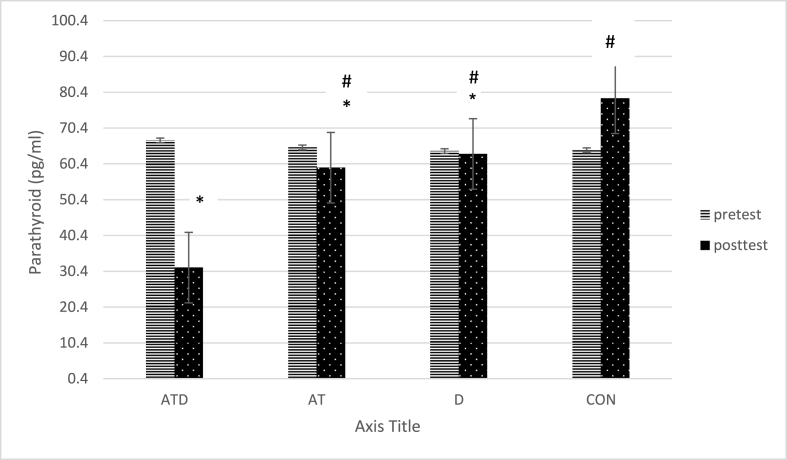
Comparison of PTH in the study groups* significant difference with CON group, # significant difference with ATD group, & significant difference with AT group, $ significant difference with D group. Aquatic training group + vitamin D intake group (ATD), Aquatic training group (AT), vitamin D intake group (D), control group (CON).

As indicated in Table 1, paired group comparisons indicated that the level of PTH in the ATD was lower compared to AT (p < 0.001), D (p < 0.001), and CON(p < 0.001) groups. Also, PTH was lower in the AT compared to the CON (D < 0.001). It was lower in the D compared to the CON (p < 0.001) (Fig. 3). There was no significant difference between other paired groups(p > 0.05).

## 4 Discussion

The findings of the study indicated that ATD increased femur BMD compared to all groups, increased serum 25(OH)D compared to AT, and control groups and reduced PTH compared to all groups. AT increased femur BMD compared to control, reduced PTH compared to control, and did not induce a significant effect on serum 25(OH)D compared to the CON group. Vitamin D supplementation just increased serum 25(OH)D compared to control and AT groups, while did not induce a significant effect on femur BMD and PTH compared to CON group. Reduction of BMI in aquatic training groups (ATD and AT) indicate sufficient effect of training program for increasing energy expenditure.

Considering BMD, AT increased BMD compared to D and CON groups. Consistent with our findings some studies have confirmed the positive effect of different exercises such as12 weeks of HIIT and moderate intensity training.^31^ Regarding the effect of exercise on menopausal women, there are controversies in findings that may be attributed to different exercise protocols.^32^ Limited studies are available about the effect of aquatic exercise on BMD. A study indicated that aquatic high intensity exercise prevented the reduction of femoral trochanter BMD.^33^ Another study found that 11 months of combined water and land-based exercise increased BMD.^34^ In the presented study when just aquatic exercise was performed as in AT group, we can conclude that 12 weeks of aquatic training reduced the rate of osteopenia and although it was effective on femur BMD compared to CON and D groups, its effect was significantly less than ATD. In other words, the combination of aquatic training with vitamin D supplementation increases their effect on femur BMD.

As this study found, the combination of aquatic training with vitamin D supplementation increased significantly femur BMD in ATD group compared to AT and CON groups. So, it seems that the combination of vitamin D with aquatic training, induces enhancing effect on femur BMD. In contrast to our findings, a descriptive study indicated that BMD in young healthy adults is associated with physical exercise, independent of sex and 25[OH]D status.^35^ Another study found that resistance exercise in combination with vitamin D and calcium had a positive effect on BMD in rats.^36^ The bone health effect of swimming can be related to adaptation in tissue-level properties, and independent of geometric changes, can be associated with the tissue composition, including increases in collagen cross-linking and changes in the carbonate/phosphate ratio.^37^

Considering the effect of vitamin D supplementation on BMD, we found no significant effect compared to the CON group. A meta-analysis review study also indicated that vitamin D supplements are not beneficial in children and adolescents with normal serum 25(OH)D levels and suggested that vitamin D supplementation of just deficient children and adolescents could result in significant improvements.^38^ In younger postmenopausal women (age: 56 y) whose average baseline serum 25(OH)D concentration was well within the normal range, the addition of 10 000 U vitamin D(2)/wk to calcium supplementation at 1000 mg/d did not enhance benefits on BMD beyond those achieved with calcium supplementation alone.^39^ In summary, it seems that the combination of vitamin D and aquatic training induces a synergic effect on increasing BMD, while vitamin D and aquatic exercise are not sufficient alone at least during 12 weeks.

Other findings of the study considering serum 25(OH)D indicated that aquatic training combined with vitamin D increased serum 25(OH)D compared to AT and CON groups, and the serum 25(OH)D level in the D group was more than the CON and AT group. Usually, sunlight exposure and vitamin D intake through supplements or nutrition affect the status of serum vitamin D. However, some studies have indicated that exercise can impact serum 25(OH)D levels. In a review study, it was shown that endurance exercise can increase serum 25(OH)D levels in people with vitamin D deficiency but was not effective in people with sufficient vitamin D. However, resistance training did not induce a significant effect. Exercise could impact serum 25(OH)D by mobilizing either the vitamin D metabolites in tissues or using it by tissues. The effects of exercise on serum 25(OH)D may be related to several factors, such as exercise mode and intensity.^40^

Similar to our findings, a study found that physical activity is not related to serum 25(OH)D,^41^ while some other studies have found that serum 25(OH)D status is related to physical activity/exercise,^42^ and exercise training impact positively serum 25(OH)D levels.^43^ However, there are discrepancies in the findings of interventional studies which may depend on the exercise mode.^27^ Studies comparing professional athletes with non-athletes found that in male runners with and without vitamin D supplementation, serum 25(OH)D was significantly increased immediately and 24 h after an ultra-marathon competition.^44^ Also, in male soccer players, serum 25(OH)D increased at 15 min and 1 h after incremental exercise.^45^ Various studies evaluated the effect of endurance training on serum 25(OH)D levels and did not show consistent results. Similar to our findings, some studies found that chronic endurance exercise training can significantly increase serum 25(OH)D levels,^46^^,^^47^ while others have found opposite results.^43^^,^^48^^,^^49^

Several factors may mediate the effect of exercise on serum vitamin D such as the vitamin D nutritional status, exercise type and intensity, and sex.^40^ Aquatic exercise can be a type of resistance exercise and similar to our findings, it has been indicated that resistance exercises caused no significant effects on serum 25(OH)D levels in people with deficient levels.^50^ We found the combination of vitamin D with aquatic training could increase serum 25(OH)D which may indicate the enhancing effect of exercise. In confirmation of our findings, another study found that endurance training combined with vitamin D intake increased serum 25(OH)D levels.^51^ In contrast to our finding, a study found that endurance exercise caused the reduction of serum 25(OH)D levels in postmenopausal women with sufficient vitamin D levels. The study was performed in late autumn and had no control group and this finding may be attributed to a seasonal decline.^48^ Our study was also conducted during late autumn and winter and serum 25(OH)D was reduced in the control and aquatic training groups, while aquatic training combined with vitamin D and vitamin D alone increased serum vitamin D. In our study aquatic training was performed in indoor swimming pool and so the effect of sun exposure during exercise is also omitted. So, we can conclude that vitamin D consumption is necessary for increasing serum 25(OH)D, and aquatic training performed in the indoor pool without sun exposure cannot compensate vitamin D intake.

Aerobic exercise, especially submaximal aerobic exercise, enhances fat metabolism and lipolysis. Adipose tissue is one of the important deposits of vitamin D.^52^ It has been suggested that the release of stored vitamin D in adipose tissue occurs during lipolysis.^53^ Lipolysis is mediated by some factors including brain natriuretic peptides (BNPs), atrial natriuretic peptides (ANPs), insulin, and adrenergic hormones.^54^ Aerobic exercise can enhance the section of these hormones^55^ to induce lipolysis which is accompanied by releasing vitamin D stored in the adipose tissue. However, according to our findings, it seems that 12 weeks of aquatic training could not induce a chronic effect on serum 25(OH)D and possibly mentioned physiological pathways.

Considering PTH, the combination of aquatic training with vitamin D reduced significantly PTH in ATD compared to the AT, D and CON groups. Also, PTH was lower in AT, and D groups compared to the CON group.

Exercise can affect PTH release by changing the serum levels of calcium and phosphate and exercise-induced myokines.^56^ PTH is expressed and released by four parathyroid glands, and control calcium homeostasis.^57^ There are controversies regarding the effect of exercise on PTH. It was found that PTH increases during a session of exercise while decreasing during the recovery period.^58^ While, it has been found that the effect of exercise training on PTH depends on vitamin D status.^59^

Our finding indicated that aquatic training combined with vitamin D decreased PTH, while increased serum vitamin D. In confirmation of our findings, another study found that in postmenopausal women, a 12-week Nordic walking exercise program increased serum 25(OH)D, while decreased PTH.^60^ It was found that PTH levels were lower in premenarcheal girls who had high physical activity than in their low-level physical activity peers.^61^ In contrast to our findings, a 8-week repeated sprint training program had no effect on circulating PTH levels.^62^ Also, 13 weeks of military training did not induce changes in PTH levels,^63^ while 6 weeks of training programs increased circulating PTH levels in the mice model.^64^ Other studies also found that vitamin D supplementation in postmenopausal women increased circulating 25(OH)D levels while decreasing serum levels of PTH significantly.^65^ The dosage of vitamin D supplementation may be effective on controversies in findings. A meta-analysis indicated that doses of 1000 IU optimally suppressed serum PTH levels, while doses of 4000 IU increased serum 25(OH)D levels in the overweight and normal obese population.^66^ However, in our study 4000IU/d was used by participants, while PTH was lower in vitamin D supplementation groups compared to the control group. It is possible that the status of serum 25(OH)D in previous studies mediates the effect of vitamin D supplementation on serum vitamin D. Another study found that vitamin D supplementation was not effective on PTH and concluded that the reason for the lack of PTH suppression can be related to the high prevalence of vitamin D insufficiency at baseline so that vitamin D supplementation could not increase the serum 25(OH)D level to cause PTH suppression.^67^ So, it can be concluded that in the present study both of aquatic training and vitamin D3 intake were effective on PTH and the lowest level of PTH in ATD group can be attributed to the enhancing effect of combining aquatic training with vitamin D3 supplementation.

### 4.1 Strength and limitations

The strength of this study was its practical nature. As it evaluated the effect of aquatic exercise and vitamin D supplementation as usually recommended exercise for elders who usually are obese and suffer from vitamin D insufficiency. A limitation of this study was the small number of participants which may impact our findings. Another limitation of this study was lack of sctict control of lifestyle factors such as nutrition, sunlight exposure, and daily physical activity of participants, although it was recommended that participants continue their usual lifestyle except for the study interventions.

## 5 Conclusions

According to our findings, the combination of aquatic training with vitamin D3 supplementation can increase femur BMD which is accompanied by increasing serum 25(OH)D and decreasing PTH. Aquatic training without vitamin D supplementation increased femur BMD which was accompanied by decreasing PTH and no significant change of serum (OH)D. Vitamin D supplementation could not induce a significant effect on BMD, although increased serum 25(OH)D, and decreased PTH. So, combination of aquatic training with vitamin D intake can be recommended as the most effective treatment to increase BMD in postmenopausal obese women with vitamin D insufficiency or lower deficiency. Also, this may rise the local effect of aquatic training which needs further studies.

## Funding

The authors received no financial support for the research, authorship, and/or publication of this article.

## Author statement

All authors agree with the content of the manuscript and approve of its submission to the Journal of Exercise Science &Fitness (JESF). We confirm that this work is original and has not been published elsewhere, nor is it currently under consideration for publication elsewhere.

## Availability of data and materials

Data are available and will be dedicated according to reasonable request.

## Declaration of competing interest

No potential conflict of interest was reported by the author(s).
